# COVID-19 Related Distress in Gambling Disorder

**DOI:** 10.3389/fpsyt.2021.620661

**Published:** 2021-02-25

**Authors:** Luana Salerno, Stefano Pallanti

**Affiliations:** ^1^INS, Istituto di Neuroscienze, Florence, Italy; ^2^Department of Psychiatry and Behavioral Sciences, Albert Einstein College of Medicine, New York, NY, United States

**Keywords:** COVID, gambling, stress, social isolation, hostility, occupation

## Abstract

The COVID-19 pandemic has exerted a dramatic impact on everyday life globally. In this context, it has been reported that the lockdown and social distancing may have exerted an impact even on gambling behavior, not only by increasing gambling behavior in those affected by this disorder but even contributing to the occurrence of new cases. To explore such a possibility, we designed a cross-sectional web survey addressing a general population sample that lasted 3 weeks (March 23–April 20). Participants completed a survey including a demographic information section, a question regarding the presence of pathological gambling in the past and several questionnaires. These included the Perceived Stress Scale (PSS), the Kellner's Symptom Questionnaire (SQ), and the version of The Yale Brown Obsessive Compulsive Scale adapted for Pathological Gambling (PG-YBOCS) that investigated the presence of gambling behaviors in the last week. The final sample was composed by 254 subjects (112 males, 44.1%; 142 females, 55.9%). According to PG-YBOCS total score, pathological gambling has been found in 23.6% (*n* = 60) of the sample (53 males, 88.3%; 7 females, 11.7%), which is a high frequency compared to that reported by the existing literature. Among gamblers, 20.9% (*n* = 53) reported both past and current problem gambling (they have been defined as “chronic gamblers”), whereas 2.8% (*n* = 7) did not report to use gambling platforms in the past but only in the last week (defined as “new gamblers”). Data analysis showed a statistically significant difference between gamblers and people who do not gamble in age but not in education, and higher level of perceived stress, distress, and hostility in both chronic and new gamblers compared to those who did not report gambling behavior. A consistent proportion of business owners and unemployed individuals reported problem gambling during the lockdown period.

## Introduction

The DSM-5 has recognized Gambling Disorder (GD) as a Substance-Related and Addictive Disorder because of the increasing evidence supporting the presence of similarities between pathological gambling and substance addiction (1, 2). GD is conceptualized as a persistent and recurrent problem gambling behavior characterized by increased tolerance and inability to stop such a behavior, which causes significant impairment and distress (3).

According to epidemiological data, the prevalence of GD ranges between 1.2 and 7.1% in the general population (4), and it seems to be higher among young people, ranging between 6 and 9% (5). A more recent systematic review reported that 0.1–5.8% of individuals meet diagnostic criteria for problem gambling across five continents during the year before the survey, whereas 0.7–6.5% meet criteria for problem gambling during their lifetime (6). A recent study performed in Italy showed low-risk gambling behavior in less than 15%, a moderate-risk in 4% and problem gambling in 1.6% (7). The use of internet seems to play a role in the rise of problem gambling, as recent evidence reported that replacing 10% of offline with online gambling increases the likelihood of being a problem gambler by 8.8–12.6%, with an increase of 27.24 million euros per year of additional expenditures (8).

The COVID-19 pandemic has exerted a dramatic impact on everyday life globally. Several studies performed in different countries around the world have reported psychological and mental health problems due to the changes caused by the COVID-19, including stress, anxiety, and depressive symptoms (9–11). According to recent data, the lockdown and social distancing may have exerted an impact even on gambling behavior (12), not only by increasing gambling behavior in those affected by this disorder but even contributing to the occurrence of new cases (13). Italy was one of the first European countries to be affected by the COVID-19 crisis, and government regulations imposed many restrictions. The latter have concerned not only individuals, who have been told to remain in their houses, but even many businesses with dramatic consequences on many persons who have not been able to work because they were unable to do their job from home (i.e., smart working). Indeed, among the limitations imposed by Italian government, it should also be mentioned the closing of retail shops different from food shops and those providing essential services (such as health ones), the suspension of the sports events and the closure of gambling and bingo halls as well as betting shops.

In consideration of data from a general population survey reported by Hakansson (14) demonstrating that a non-negligible percentage of respondents reported an increase of gambling behavior during the COVID-19 pandemic, we aimed to investigate if there was a similar increase in Italian population during the lockdown period, and if there were some differences in demographic as well psychological variables (e.g., perceived stress, distress, anxiety, depression, well-being) between those who had gambling problems and those who did not. For this aim, we designed a cross-sectional web survey addressing a general population sample that lasted 3 weeks (March 23–April 20).

## Methods

### Design and Participants

This is a cross-sectional web survey addressing a general population sample. We recruited participants using ads on facebook groups and information pages regarding the Italian situation relating to COVID-19, psychology, physical and mental health on other social media channels (i.e., twitter, telegram, instagram). The participants were also invited to in turn forward the invitation onto their own facebook/other social media friends. They were all over 18 years of age and where able to open the survey only after receiving the study information; on the first page, they were asked to give their consent to study participation. The study was carried out during a period of 3 weeks (from March 23 to April 20). The survey did not include any information that could directly or indirectly identify an individual. Researchers could not detect the IP-addresses. No compensation to take part to the study was provided. As the study involved human subjects, it was conducted in accordance with the Declaration of Helsinki.

### Measures

Basic socio-demographic variables included age, gender and occupation. After an explanation on what was considered as “gambling” (i.e., gambling for money through online platforms including betting sites, casinos, poker games, lotteries, bingo etc., and through the corresponding on-land based counterparts, even including slot machines and instant lotteries), respondents had to report if they have used gambling online and on-land platforms in the past 3 years or if they had started using them since the lockdown beginning. On this basis, they have been classified as “chronic gamblers” if they reported a past use of gambling platforms, with a need to gamble with increasing amount of money, restlessness or irritability when trying to cut down or stop gambling, had repeated unsuccessful efforts to control, cut back on or stop gambling in the last 3 years, “new gamblers” if they reported a beginning during the lockdown period, and “no gamblers” if they have reported they never used these platforms.

The severity of pathological gambling within the past week has been assessed by the Pathological Gambling Adaptation of Yale-Brown Obsessive Compulsive Scale (PG-YBOCS) (15). The first five questions assess urges and thoughts associated with gambling, whereas the last five questions assess the behavioral component of the disorder. The sum score of each subscale ranges from 0 to 20. Each subscale can be analyzed separately as well as together as a total score. The total score can be interpreted as follows: 0–7 sub-clinical, 8–15 mild, 16–23 moderate, 24– 31 severe, and 32–40 extreme gambling symptoms. Originally the questionnaire was used as a semi-structured interview, however, in the present study the PG-YBOCS was administered as an online self-rating questionnaire, which is expected to be unproblematic as both versions (interview and self-rating) show good convergent validity for the YBOCS (16). With regard to construct validity, the PG-YBOCS and its two subscales correlated moderately strongly with the SOGS, which is a reliable screening instrument for pathological gambling based on the DSM-IV diagnostic criteria and a suitable measure of lifetime gambling severity (15). Moreover, PG-YBOCS showed good content validity in severity and change highly correlated with SOGS (15).

As a measure of perceived stress, the Perceived Stress Scale (PSS) developed by Cohen, Kamarck and Mermelstein (17) has been administered. It is a well-established self-report measure assessing “the degree to which situations in one's life are appraised as stressful” [(17), p. 387], and the degree to which life has been experienced as unpredictable, uncontrollable, and overloaded in the past month.

For the assessment of psychological symptoms (depression, anxiety, hostility, and somatization) and well-being (contentment, relaxation, friendliness, and physical well-being) we used the Symptom Questionnaire (SQ), which is a simple, self-rated questionnaire developed by Robert Kellner in 1976 (18). The final version of the SQ consists of 92 items and yields four main scales: depression, anxiety, hostility, and somatization. Each scale can be divided into two subscales, one concerned with symptoms and the other with well-being, for a total of eight subscales. Therefore, each of the main scales includes items from both the symptoms and the well-being subscales. Answers are dichotomous, and the respondent is asked to check YES/NO or TRUE/FALSE for each item. Scales and subscales can be scored separately, and the sum of the four main scale scores yields a total distress score. Two forms of the SQ are available (week and daily form). In this study we used the week form that is concerned with feelings experienced by the respondent during the past week. We considered the four main scale scores as well as the well-being subscales to investigate the presence of some associations between these dimensions and gambling.

### Statistical Analysis

Statistical analyses were performed with the Statistical Package for the Social Sciences version 23.0 (SPSS; IBM Corp., 2015). Descriptive analyses have been reported as means, percentage and medians. For what concerns comparisons between groups regarding psychological measures, since data were not normally distributed as assessed by visual inspection of the boxplots, the Kruskal-Wallis *H*-test (sometimes also called the “one-way ANOVA on ranks”) has been used to determine if there were statistically significant differences between chronic, new and no gamblers, while to compare chronic vs. new gamblers we used the Mann-Whitney *U*-test. Statistical significance was set at *p* < 0.05, two-tailed.

## Results

A total of 316 subjects were able to open the survey after receiving the study information, but only 281 gave their consent to participate in the study. Of these, 27 left the survey incomplete and were therefore excluded from the analysis through listwise deletion.

The final sample was composed by 254 subjects (112 males, 44.1%; 142 females, 55.9%) with a mean age of 33.65 ± 13.21. There was not a statistically significant differences among the number of participants recruited from the different social channels [*n* = 135 from Facebook, *n* = 119 from the other channels, $\chi_{\left(1\right)}^{2}$ = 0.069, *p* = 0.882]

According to PG-YBOCS total score, pathological gambling has been found in 23.6% (*n* = 60) of our sample (53 males, 88.3%; 7 females, 11.7%). Among gamblers, 20.9% (*n* = 53) reported past and current problem gambling (and therefore they have been defined as “chronic gamblers”), whereas 2.8% (*n* = 7) did not report to gamble in the past but only in the last week (they have been classified as “new gamblers”). There was a statistically significant difference among groups (no gamblers, chronic and new gamblers). Indeed, no gamblers were predominantly females (135 vs. 59), whereas chronic gamblers were predominantly males (46 vs. 7) and new gamblers were all males (7 vs. 0) [$\chi_{\left(2\right)}^{2}$ = 62.804, *p* < 0.001].

For what concerns occupation, 30.7% of the total sample was mainly composed by students (30.7%) followed by healthcare practitioners (20.1%) and people working in the field of administrative support (13.8%). Interestingly, the chronic gamblers were predominantly business owners, people who worked in the administrative support field, unemployed and people who worked in the production sector. Instead, new gamblers were mostly unemployed (71.4%) and business owners (28.6%). Tables 1, 2 show types of occupation and business ownerships according to the groups.

**Table 1:** Types of occupation according to groups.

**Occupation**	**Total (*N* = 254)** ***n* (%)**	**No gamblers (*N* = 194)** ***n* (%)**	**Chronic gamblers (*N* = 53)** ***n* (%)**	**New gamblers (*N* = 7)** ***n* (%)**
Business owner	25 (9.8%)	11 (5.7%)	12 (22.6%)	2 (28.6%)
Physician	2 (0.8%)	2 (1%)	–	–
Healthcare practitioner	51 (20.1%)	48 (24.7%)	3 (5.7%)	–
Student	78 (30.7%)	77 (39.7%)	1 (1.9%)	–
Arts and design	6 (2.4%)	4 (2.1%)	2 (3.8%)	–
Police	1 (0.4%)	1 (0.5%)	–	–
Unemployed	15 (5.9%)	3 (1.5%)	7 (13.2%)	5 (71.4%)
Retired	3 (1.2%)	3 (1.5%)	–	–
Legal	4 (1.6%)	2 (1%)	2 (3.8%)	–
Sales	9 (3.5%)	5 (2.6%)	4 (7.5%)	–
Administrative support	35 (13.8%)	25 (12.9%)	10 (18.9%)	–
Education	6 (2.4%)	3 (1.5%)	3 (5.7%)	–
Engineering	8 (3.1%)	4 (2.1%)	4 (7.5%)	–
Production	11 (4.3%)	6 (3.1%)	5 (9.4%)	–

**Table 2:** Types of business ownership according to groups.

**Sectors**	**No gamblers** **(*n*, %)**	**Chronic gamblers** **(*n*, %)**	**New gamblers** **(*n*, %)**
Retail business	5 (20.0%)	3 (12.0%)	1 (4.0%)
Restaurants/nightclubs	2 (8.0%)	5 (20.0%)	1 (4.0%)
Travel agency	0 (0.0%)	2 (8.0%)	0 (0.0%)
Construction company	0 (0.0%)	1 (4.0%)	0 (0.0%)
Transportation business	0 (0.0%)	1 (4.0%)	0 (0.0%)
Fashion business	1 (4.0%)	0 (0.0%)	0 (0.0%)
Service business	3 (12.0%)	0 (0.0%)	0 (0.0%)

**Figure 1:**
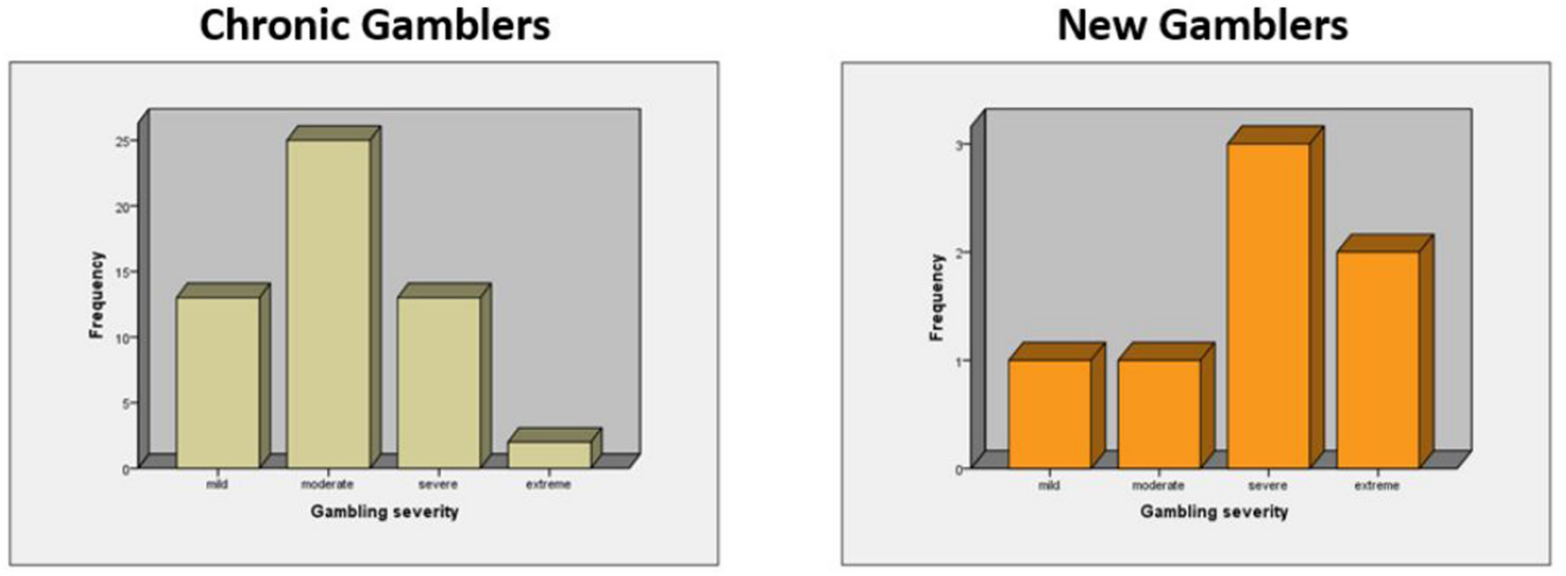
Gambling behavior severity among chronic and new gamblers.

As Figure 1 shows, according to the PG-YBOCS scores, the severity of gambling in chronic gamblers was mild in 24.5%, moderate in 47.2%, severe in 24.5%, and extreme in 3.8% of them. Gambling severity was mild and moderate in 14.3% of new gamblers and severe and extreme in 42.9 and 28.6% of them, respectively.

A Kruskal-Wallis *H*-test was run to determine if there were differences in age, education, PSS and SQ scores among the three groups of participants: the “no gambling,” the “chronic” and the “new gamblers.” Age were lower for no gamblers compared to new and chronic gamblers [$\chi_{\left(2\right)}^{2}$ = 47.354, *p* = <0.001]; even though education level was similar across no gamblers and new gamblers and lower in chronic gamblers, the differences were not statistically significant [$\chi_{\left(2\right)}^{2}$ = 3.823, *p* = 0.148].

For what concerns PSS and SQ scores, those who did not report to gamble obtained lower PSS compared to chronic and new gamblers [$\chi_{\left(2\right)}^{2}$ = 67.090, *p* = <0.001], lower SQ Anxiety compared to chronic and new gamblers [$\chi_{\left(2\right)}^{2}$ = 102.078, *p* = <0.001], lower SQ Depression compared to chronic and new gamblers [$\chi_{\left(2\right)}^{2}$ = 69.834, *p* = <0.001], lower SQ Somatization compared to chronic and new gamblers [$\chi_{\left(2\right)}^{2}$ = 46.719, *p* = <0.001], lower SQ Hostility compared to chronic and new gamblers [$\chi_{\left(2\right)}^{2}$ = 52.324, *p* = <0.001], and lower SQ Distress scores compare to new gamblers and chronic gamblers [$\chi_{\left(2\right)}^{2}$ = 97.871, *p* = <0.001].

For what concerns SQ well-being scale and subscales scores, people who have never gambled showed higher scores at SQ Physical well-being scale compared to chronic and new gamblers [$\chi_{\left(2\right)}^{2}$ = 67.972, *p* = <0.001], SQ Relaxation subscale compared to chronic and new gamblers [$\chi_{\left(2\right)}^{2}$ = 89.773, *p* = <0.001], SQ Contentment subscale compared to chronic and new gamblers [$\chi_{\left(2\right)}^{2}$ = 57.949, *p* = <0.001], and SQ Friendliness subscale compared to chronic and new gamblers [$\chi_{\left(2\right)}^{2}$ = 20.791, *p* = <0.001].

The Mann-Whitney *U*-test was carried out as a *post-hoc* test for pairwise comparisons (Bonferroni correction) and results showed no statistically significant differences in the PSS and SQ scales and subscale scores (*p* > 0.05). Medians are reported in Table 3.

**Table 3:** Differences in median values between groups.

	**No gamblers** **(*n* = 194)**	**Chronic gamblers** **(*n* = 53)**	**New gamblers** **(*n* = 7)**	
Age	25	42	36	*p* = <0.001
Education	15	13	15	*p =* 0.148
PSS	19	25	25	*p* = <0.001
SQ Anxiety	7	18	18	*p* = <0.001
SQ Depression	5.50	12	12	*p* = <0.001
SQ Somatization	7	14	14	*p* = <0.001
SQ Hostility	5.50	12	15	*p* = <0.001
SQ Physical well-being	3	1	1	*p* = <0.001
SQ Relaxation	4	1	0	*p* = <0.001
SQ Contentment	4	1	1	*p* = <0.001
SQ Friendliness	4	1	1	*p* = <0.001
SQ Distress	25.50	55	57	*p* = <0.001

## Discussion

The present study investigated the impact of social-distancing during the COVID-19 pandemic on gambling behavior in a sample of Italian individuals. At the time of this writing, the cases of COVID-19 in Italy were 318.065, with 35.992 deaths and 221.867 healed (https://www.epicentro.iss.it/coronavirus/sars-cov-2-dashboard). Italy was the first nation in Europe affected by COVID-19, and because of its rapid spread and dangerousness the lockdown was considered the only means to protect people, especially the most vulnerable, reorganize resources, and to give hospitals time to organize optimal care. In Italy the lockdown started on March 9th and ended on May 18th, and our survey begun 2 weeks after the starting of social isolation. Our data show that in that period 23.6% of individuals suffered from pathological gambling, a frequency that is much higher than that generally reported, they were more frequently male (88.3 vs. 11.7%), and many of them were unemployed or business owners. Even though it is not clear if such work situations as precariousness or unemployment play a role in the development and/or maintenance of gambling behavior (19, 20), it is important to consider that during the lockdown period hospitality and travel industry was hit hard, as were the owners of restaurants and clubs who had to close, with the concern that they could no longer bear the costs of running their business. In fact, what was then a concern turned out to be a reality, with many of them finding themselves unable to reopen due to the reduction in tourism and the inability to meet operating costs (https://www.thelocal.it/20200522/italys-shops-and-restaurants-struggle-to-reopen-with-new-rules-and-lack-of-customers). Some of them attempted suicide (https://www.theguardian.com/world/2020/may/21/italy-lockdown-mental-health-psychologists-coronavirus). In our sample, business owners with chronic gambling behavior were predominantly owners of restaurants/nightclubs, retail business and travel agency, while two out of five of new gamblers were owners of retail business and restaurants/nightclubs. The high number of unemployed in new gamblers group is in line with evidence suggesting that potentially problem or at-risk gamblers have difficulty in money management and are used to spend more than they earn (21, 22).

In our sample, both chronic and new gamblers obtained higher scores at measures of perceived stress, anxiety, depression, somatization, hostility, and distress compared to those who never gambled, and lower scores at measures of well-being. These findings are in line with literature reporting gambling as a means to cope with negative emotions in people characterized by high psychological distress and as associated with a higher likelihood of reporting problems related to multiple life domains, including hostility, and aggressiveness (23).

Our study confirmed findings from Hakansson (14) indicating a trend for the appearance of new gamblers during social-distancing caused by the COVID-19 pandemic. Although we do not know if this is a consequence of concerns about money or of the increase on the amount of time spent at home leading to more time spent online (24), prior national or international financial crises have been reported to have had an influence on gambling behaviors and on exacerbation of gambling problems (25), including the financial crisis in Greece (26) and in Iceland (27).

The findings of this study have to be seen in light of some limitations. First of all, our study is an anonymous web survey and not a face-to-face interview, which even though was the only way to collect data during the COVID-19 lockdown period it did not let us to collect more in-depth data nor investigate the types, patterns and severity of past gambling behaviors. Secondly, we did not use a screening tool for pathological gambling such as the SOGS, but even though our data may not be considered as an estimated prevalence, as previously reported the two subscales of PG-YBOCS showed a moderately strong correlation with the SOGS (15). Third, we did not assess the presence of pre-existing psychological vulnerability factors, other medical issues such as chronic illness making subjects more at risk of severe COVID, or co-existent psychiatric conditions such as substance use disorders (e.g., alcohol, pain killer etc.). Fourth, we did not collect data on family composition or on the presence of children who, given the closure of schools, were forced to stay at home all day, causing a possible increase in stress. Finally, we used different channels for recruitment for assuring a representative non-clinical population recruited by social media, but these findings may not be the same in the “real world.”

Although the study limitations, our findings indicated a consistent proportion of business owners and unemployed individuals who reported pathological gambling during the lockdown period, and a higher level of perceived stress, distress and hostility in both chronic and new gamblers compared to those who never reported gambling behavior. As the prospect theory by Kahneman and Tvesky (28) demonstrated, agents are more sensitive to losses than to gains and even the small chance of a large win can seem very alluring. According to the prospect theory, as losses accumulate, subjects could become more willing to take additional risk, and they could therefore persevere in gambling. In the context of the economic crisis caused by the COVID-19, and considering the high availability of online gambling platforms, rapid actions for regulatory measures and prevention by multiple stakeholders are necessary.

## Data Availability Statement

The original contributions presented in the study are included in the article, further inquiries can be directed to the corresponding author.

## Ethics Statement

Ethical review and approval was not required for the study on human participants in accordance with the local legislation and institutional requirements. The participants provided their written informed consent to participate in this study.

## Author Contributions

LS and SP contributed to the conception and design of the study. LS undertook the statistical analysis and wrote the first draft of the manuscript. All the authors contributed to manuscript revision, read, and approved the submitted version.

## Conflict of Interest

The authors declare that the research was conducted in the absence of any commercial or financial relationships that could be construed as a potential conflict of interest.

## References

[1] Grant JE Potenza MN Weinstein A Gorelick DA. Introduction to behavioral addictions. Am J Drug Alcohol Abuse. (2010) 36:233–41. 10.3109/00952990.2010.491884 20560821 PMC3164585

[2] Blum K Febo M McLaughlin T Cronjé FJ Han D Gold SM. Hatching the behavioral addiction egg: Reward Deficiency Solution System (RDSS)™ as a function of dopaminergic neurogenetics and brain functional connectivity linking all addictions under a common rubric. J Behav Addict. (2014) 3:149–56. 10.1556/JBA.3.2014.019 25317338 PMC4189308

[3] American Psychiatric Association. Diagnostic and Statistical Manual of Mental Disorders. 5th ed. Arlington: American Psychiatric Association (2013).

[4] Conversano C Marazziti D Carmassi C Baldini S Barnabei G Dell'Osso L. Pathological gambling: a systematic review of biochemical, neuroimaging, and neuropsychological findings. Harv Rev Psychiatry. (2012) 20:130–48. 10.3109/10673229.2012.694318 22716504

[5] Barnes GM Welte JW Hoffman JH Tidwell M-CO. Comparisons of gambling and alcohol use among college students and noncollege young people in the United States. J Am Coll Health. (2010) 58:443–52. 10.1080/07448480903540499 20304756 PMC4104810

[6] Calado F Griffiths MD. Problem gambling worldwide: an update and systematic review of empirical research (2000-2015). J Behav Addict. (2016) 5:592–613. 10.1556/2006.5.2016.073 27784180 PMC5370365

[7] Benedetti E Molinaro S Potente R Scalese M Siciliano V Luppi C. I dati sul gioco d'azzardo in Italia. In: Coordinamento Nazionale Comunità Accoglienza (CNCA). Year Book 2016. Roma: Comunità Edizioni. (2016). p. 26–44.

[8] Effertz T Bischof A Rumpf H-J Meyer C John U. The effect of online gambling on gambling problems and resulting economic health costs in Germany. Eur J Health Econ. (2018) 19:967–78. 10.1007/s10198-017-0945-z 29362900

[9] Zeppegno P Gramaglia C Guerriero C Madeddu F Calati R. Psychological/psychiatric impact of the novel coronavirus outbreak: lessons learnt from China and call for timely crisis interventions in Italy. PsyArXiv. (2020). 10.31234/osf.io/z26yk

[10] Karpenko OA Syunyakov TS Kulygina MA Pavlichenko AV Chetkina AS Andrushchenko AV. Impact of COVID-19 pandemic on anxiety, depression and distress – online survey results amid the pandemic in Russia. Consortium Psychiatricum. (2020) 1:8–20. 10.17650/2712-7672-2020-1-1-8-20 PMC11047270 38680383

[11] Alyami HS Naser AY Dahmash EZ Alyami MH Al Meanazel OT Al-Meanazel AT. Depression and anxiety during 2019 coronavirus disease pandemic in Saudi Arabia: a cross-sectional study. medRxiv. (2020). 10.1101/2020.05.09.2009667732578943

[12] Håkansson A Fernández-Aranda F Menchón JM Potenza MN Jiménez-Murcia S. Gambling during the COVID-19 crisis - a cause for concern? J Addict Med. (2020) 14:e10–2. 10.1097/ADM.0000000000000690 32433365 PMC7273946

[13] Yahya AS Khawaja S. Problem gambling during the COVID-19 pandemic. Prim Care Companion CNS Disord. (2020) 22:20com02690. 10.4088/PCC.20com02690 32731317

[14] Håkansson A. Changes in gambling behavior during the COVID-19 pandemic—a web survey study in Sweden. Int J Environ Res Public Health. (2020) 17:1–16. 10.3390/ijerph17114013 32516880 PMC7312016

[15] Pallanti S DeCaria CM Grant JE Urpe M Hollander E. Reliability and validity of the pathological gambling adaptation of the Yale-Brown Obsessive-Compulsive Scale (PG-YBOCS). J Gambl Stud. (2005) 21:431–43. 10.1007/s10899-005-5557-3 16311876

[16] Goodman WK Price LH Rasmussen SA Mazure C Delgado P Heninger GR. The yale-brown obsessive compulsive scale. Arch Gen Psychiatry. (1989) 46:1006. 10.1001/archpsyc.1989.01810110048007 2510699

[17] Cohen S Kamarck T Mermelstein R. A global measure of perceived stress. J Health Soc Behav. (1983) 24:385–96. 10.2307/2136404 6668417

[18] Kellner R. Abridged manual of the Symptom Questionnaire. Albuquerque: University of New Mexico (1976).

[19] Rolando S Beccaria F. ‘Got to gamble, but I've got no money.’ A qualitative analysis of gambling careers in South Italy. Int Gambl Stud. (2019) 19:106–24. 10.1080/14459795.2018.1517816

[20] Arge EM Kristjánsson S. The effects of unemployment on gambling behaviour in Iceland: Are gambling rates higher in unemployed populations? (Diss.) (2015). Available online at: http://hdl.handle.net/1946/21436

[21] Barbaranelli C. Prevalence and correlates of problem gambling in Italy. Paper presented at 8th European Conference on Gambling Studies and Policy Issues. Vienna (2010).

[22] Barbaranelli C Vecchione M Fida R Podio-Guidugli S. Estimating the prevalence of adult problem gambling in Italy with SOGS and PGSI. J Gambling Issues. (2013) 28:1–24. 10.4309/jgi.2013.28.3

[23] Suomi A Nicki AD Jackson AC. Problem gambling subtypes based on psychological distress, alcohol abuse and impulsivity. Addict Behav. (2014) 39:1741–5. 10.1016/j.addbeh.2014.07.023 25119420

[24] King D Delfabbro P Billieux J Potenza M. Problematic online gaming and the COVID-19 pandemic. J Behav Addict. (2020) 9:184–6. 10.1556/2006.2020.00016 32352927 PMC8939428

[25] Jiménez-Murcia S Fernández-Aranda F Granero R Menchón JM. Gambling in Spain: update on experience, research and policy. Addiction. (2014) 109:1595–601. 10.1111/add.12232 23718704

[26] Economou M Souliotis K Malliori M Peppou LE Kontoangelos K Lazaratou H. Problem gambling in Greece: prevalence and risk factors during the financial crisis. J Gambl Stud. (2019) 35:1193–210. 10.1007/s10899-019-09843-2 31165324

[27] Olasen DT Hayer T Brosowski T Meyer G. Gambling in the mist of economic crisis: results from three national prevalence studies from Iceland. J Gambl Stud. (2015) 31:759–74. 10.1007/s10899-015-9523-4 25656216

[28] Kahneman D Tversky A. Prospect theory: an analysis of decision under risk. Econometrica. (1979) 47:263–91.

